# A Practical Guide to Measuring *Ex vivo* Joint Mobility Using XROMM

**DOI:** 10.1093/iob/obaa041

**Published:** 2020-11-12

**Authors:** Armita R Manafzadeh

**Affiliations:** Department of Ecology and Evolutionary Biology, Brown University, Providence, RI 02912, USA

## Abstract

X-Ray Reconstruction of Moving Morphology (XROMM), though traditionally used for studies of *in vivo* skeletal kinematics, can also be used to precisely and accurately measure *ex vivo* range of motion from cadaveric manipulations. The workflow for these studies is holistically similar to the *in vivo* XROMM workflow but presents several unique challenges. This paper aims to serve as a practical guide by walking through each step of the *ex vivo* XROMM process: how to acquire and prepare cadaveric specimens, how to manipulate specimens to collect X-ray data, and how to use these data to compute joint rotational mobility. Along the way, it offers recommendations for best practices and for avoiding common pitfalls to ensure a successful study.

## Motivation

**Fig. 1:**
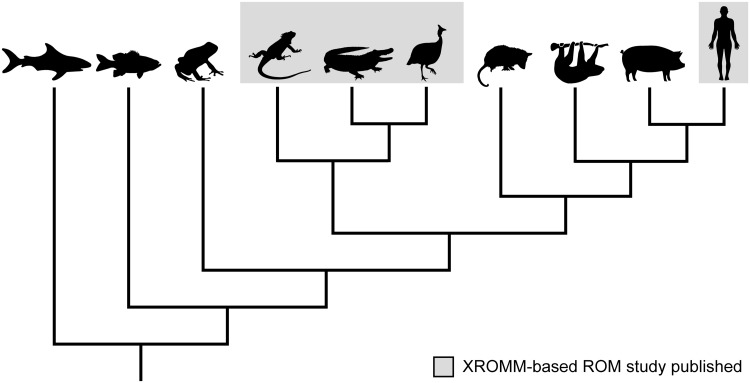
A sample of the vertebrate taxa studied to date using XROMM. *In vivo* XROMM studies have already investigated the musculoskeletal motion of a broad phylogenetic diversity of vertebrates, including (left to right) sharks, fish, toads, lizards, alligators, birds, opossums, sloths, pigs, and humans (citations in text). However, XROMM-based ROM studies have thus far only been published for lizards (green iguana [*Iguana iguana*; Arnold et al. 2014]), alligators (American alligator [*Alligator mississipiensis*; Manafzadeh et al. under review]), birds (Helmeted Guineafowl [*Numida meleagris*; Kambic et al. 2017a; Manafzadeh et al. under review], common quail [*Coturnix coturnix*; Manafzadeh and Padian 2018], wild turkey [*Meleagris gallopavo*; Kambic et al. 2017b], and humans [*Homo sapiens*; Akhbari et al. 2019]).

The advent of X-ray Reconstruction of Moving Morphology (XROMM; both marker-based [Brainerd et al. 2010] and markerless [Gatesy et al. 2010]) has revolutionized comparative biomechanists’ ability to visualize and measure musculoskeletal motion. XROMM is an X-ray motion analysis technique that integrates movement data from biplanar X-ray videos with morphological data from 3D scans of skeletal elements to create precise and accurate re-animations of vertebrate motion. Over the past decade, this methodology has earned acclaim for enabling an unprecedented understanding of the *in vivo* kinematics of skulls, ribs, and limbs from across the vertebrate tree (Fig. 1; e.g., Nyakatura and Fischer 2010; Stefen et al. 2011; Griep et al. 2013; Miranda et al. 2013; Kambic et al. 2014, 2015; Camp et al. 2015; Menegaz et al. 2015; Montuelle and Williams 2015; Bonnan et al. 2016; Brainerd et al. 2016; Panagiotopoulou et al. 2016; Fischer et al. 2018; Orsbon et al. 2018; Bhullar et al. 2019, 2020; Capano et al. 2019; Laurence-Chasen et al. 2019; Lin et al. 2019; Montuelle et al. 2019; Nyakatura et al. 2019; Olsen et al. 2019; van Meer et al. 2019; Akhbari et al. 2020; Lomax et al. 2020; Tsai et al. 2020; Weller et al. 2020). In addition, however, XROMM has also facilitated the first comprehensive measurements of joint range of motion (ROM)—the full set of poses a joint can reach—from cadaveric manipulations (Arnold et al. 2014; Kambic et al. 2017a, 2017b; Manafzadeh and Padian 2018; Akhbari et al. 2019; Manafzadeh et al. under review). 

*Ex vivo* studies of joint mobility can contribute substantially to our collective understanding of articular biomechanics. For example, manipulations of reduced cadaveric preparations can reveal how different articular structures allow and constrain motion in various taxa (e.g., Vishteh et al. 1999; Carpenter and Wilson 2008; Martin et al. 2008; Hutson and Hutson 2012, 2013, 2014, 2015a, 2015b, 2018; Pierce et al. 2012; Cobley et al. 2013; Arnold et al. 2014; Jurestovsky et al. 2020), while a growing collection of intact cadaveric manipulations will enable analyses of the development and evolution of joint mobility. Using XROMM (in contrast with traditional 2D goniometer or protractor-based methods) to conduct these studies provides biomechanists with an accurate and reproducible way to calculate all possible biologically meaningful, six-degree-of-freedom joint poses. However, it carries with it a unique set of challenges that may surprise even the most experienced XROMM user. The goal of this paper is to offer a concise, accessible, and practical guide to all aspects of measuring *ex vivo* joint mobility using XROMM.

## Overview

**Fig. 2:**
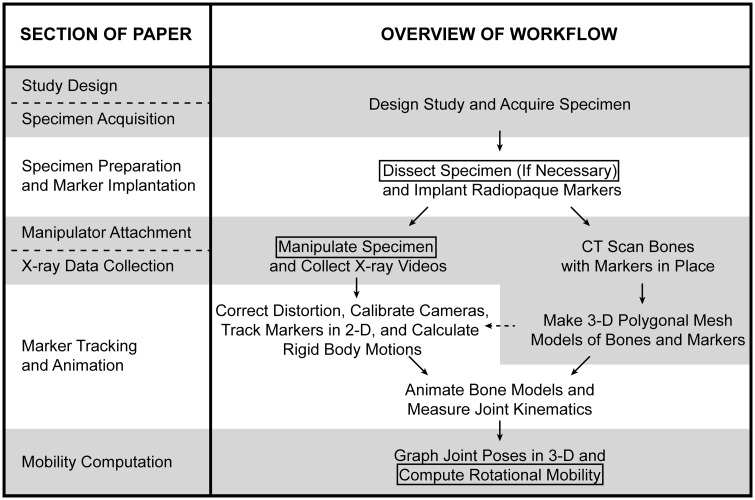
Overview of the workflow for measuring *ex vivo* joint mobility using XROMM. Modified from the *in vivo* workflow presented by Brainerd et al. (2010) and represents a marker-based study using XMALab for analysis (Knörlein et al. 2016). Aspects of the workflow that are unique to *ex vivo* studies are boxed; each section of this paper is aligned with the step(s) of the workflow that it discusses.

Holistically, the workflow for using marker-based XROMM to measure *ex vivo* joint mobility (Fig. 2) is very similar to the workflow originally outlined for *in vivo* studies by Brainerd et al. (2010). In brief: after designing the study and acquiring cadaveric specimens, implant three or more radiopaque markers into each rigid skeletal element of interest. Then use one or more wooden rods attached to the specimen to safely manipulate it inside the volume created by two X-ray image machines, and collect biplanar X-ray videos of the joint being moved through its full ROM. After correcting fluoroscope distortion, calibrating the X-ray cameras, and tracking the 2D positions of the radiopaque markers in both views, calculate rigid body transformations (RBTs). Use these RBTs to animate computed tomography (CT)-derived mesh models of the skeletal elements. Finally, measure joint poses from the resulting animation, graph them in a 3D joint pose space, and compute rotational mobility.

The aspects of this workflow that are unique to *ex vivo* studies (indicated by boxes in Fig. 2)—and therefore present unique challenges—are: (1) in some cases, tissues must be resected from the specimen to create a reduced cadaveric preparation before data collection (see Study design); (2) the specimen must be manipulated by a researcher from a safe distance outside the X-ray volume to move the joint of interest through its full ROM (see Manipulator attachment section); and (3) to compute rotational mobility, joint poses must be plotted in a 3D joint pose space rather than as the 2D angle versus time curves typically presented in XROMM papers (e.g., Kambic et al. 2014; Menegaz et al. 2015; see Mobility computation section).

## Study design

Previous cadaveric ROM studies have been conducted both using intact cadaveric specimens and using reduced preparations from which some soft tissues have been removed. When designing an XROMM ROM study, begin by thoroughly considering which of these approaches (or what combination of them) will best answer the research question at hand.

The morphology and mechanical properties of articular structures such as bone, cartilage, joint capsules, and capsular and intracapsular ligaments—as well as the muscles and tendons that cross joints and the integument that surrounds them—all influence joint ROM (Archer 1999). Therefore, only manipulations of fully intact cadavers will offer a strong *ex vivo* indicator of all the joint poses that an animal could passively assume in life (i.e., “true” mobility). Future studies should compare *in vivo* passive ROM (e.g., Hammond 2014) to intact cadaveric ROM to quantitatively validate this proxy, but especially for rare taxa, the feasibility of collecting anesthetized *in vivo* data is limited.

That said, maintaining an intact cadaver requires skilled radiopaque marker implantation (see Specimen preparation and marker implantation section) and attaching a manipulator rod to an intact joint of interest can prove difficult (see X-ray data collection section). This type of study also substantially complicates measurements of joint loading regimes, which require firm attachment of a load cell. Manipulations of reduced preparations are thus well-suited to investigations of particular tissues’ effects on ROM (e.g., “ligamentous” ROM measured with only ligaments and the joint capsule intact; e.g., Martin et al. 2008; Pierce et al. 2012; Hutson and Hutson 2012, 2013, 2014, 2015a, 2015b, 2018; Cobley et al. 2013; Arnold et al. 2014; Manafzadeh and Padian 2018) or studies that require simultaneous measurements of joint moments.

## Specimen acquisition

Obtaining cadavers can be surprisingly challenging—especially for studies on rare or endangered taxa. Whenever possible, it is ideal to repurpose individuals previously used in *in vivo* XROMM studies (e.g., Arnold et al. 2014; Kambic et al. 2017a) because the desired skeletal elements may already be surgically marked, and the resulting cadaveric data can be used in subject-specific comparisons with the *in vivo* data previously collected (see Mobility computation section). In many instances, of course, no such individuals will exist and new specimens will need to be acquired. Social media networks and meetings of scientific societies can serve as useful avenues for connecting with colleagues who may have cadavers resulting from their own *in vivo* studies. Alternatively, specimens of some species may be available for research purposes from local zoos, shelters, or wildlife rescue and rehabilitation agencies.

Before accepting specimens, ensure that they were frozen soon after death and have not been thawed since to avoid changes to the mechanical properties of joint soft tissues (see Dawson et al. 1958; Woo et al. 1986; Clavert et al. 2001; Zhang et al. 2017; though the effects of freezing and thawing on overall ROM are poorly understood). Likewise, ensure that they have been well-packed in the freezer to avoid desiccation and that no fixatives (such as formalin, which changes the mechanical properties of vertebrate tissue [Wilke et al. 1996; Hohmann et al. 2019]) have been applied. If shipping cadavers by postal service, request that they are triple bagged and sent by overnight mail, either on dry ice or in a cooler with ice packs, to prevent thawing or leakage. Note that the animal ethics or welfare committee at some institutions may require advance notice of cadaver procurement, and the transport of some species may require legal permits to be obtained at the local or national level—so confirm policies and plan accordingly before requesting shipments.

## Specimen preparation and marker implantation

Once specimens have been obtained, thaw and refreeze them as little as possible (see references discussing the effects of freezing and thawing on vertebrate tissue in Specimen Acquisition, above). Ideally, specimens should be thawed only once, and radiopaque markers should be implanted as soon as possible after thawing to limit the amount of time that the specimen is at room temperature. Either collect X-ray data immediately or tightly seal the specimen in a plastic bag, refrigerate it overnight, and collect X-ray data the following day. Record information about freeze, thaw, and refrigeration timings for each specimen; these metadata may become important once the effects of freeze–thaw cycles on joint mobility are better understood.

To create a reduced preparation (e.g., one from which all integument and muscles have been removed, but the joint capsule and ligaments are left intact), resect all desired tissues under a dissection microscope to confirm that underlying tissues are not cut. Even small, accidental incisions to a joint capsule can compromise its effects on mobility—and these defects will also grow quickly as the specimen is manipulated. Once dissection begins, make sure to regularly irrigate reduced preparations with Ringer’s solution or physiological saline to prevent desiccation.

If specimens were not already marked in a previous *in vivo* XROMM study, implant three or more radiopaque markers into each skeletal element of interest (whenever possible, implanting four or five markers is better; Brainerd et al. 2010). Full marker implantation is critical because manual scientific rotoscoping, which involves hand-aligning 3D bone models to their X-ray shadows, is not realistically feasible for the tens of thousands of joint poses that ROM studies require (see X-ray data collection section; Gatesy et al. 2010). However, if the specimen’s anatomy is well-suited to the automatic rotoscoping software Autoscoper, marker implantation may not be necessary (previously applied only to human joints and not discussed further here; see Bey et al. 2006; Miranda et al. 2011; and xromm.org for more information; and see an application to an XROMM-based ROM study by Akhbari et al. [2019]).

When selecting radiopaque markers, use spherical ball bearings (e.g., Menegaz et al. 2015) in place of conical markers (e.g., Kambic et al. 2014) whenever possible. The XROMM analysis software XMALab (Knörlein et al. 2016; see Marker tracking and animation section) detects marker positions using a circular region of interest, so spherical markers will result in lower reprojection errors and a higher calculated precision (defined as the average of standard deviations of inter-marker distances for all co-osseous pairs; see Brainerd et al. 2010). That said, see Kambic et al. (2014) for information about fabricating and implanting conical markers if the anatomy of the specimen precludes the use of spherical markers for some or all implantation sites. Likewise, select zirconium oxide makers (e.g., Manafzadeh and Padian 2018) rather than tantalum ones (e.g., Menegaz et al. 2015) whenever possible. Zirconium oxide creates much smaller starburst artifacts during CT scanning because it has a lower X-ray attenuation coefficient than tantalum (see an explanation in Neyman et al. 2002). As a result, it allows more accurate 3D reconstructions of articular geometry, which can assist in the creation of standardized and reproducible joint coordinate systems (JCSs; *sensu*Grood and Suntay 1983; see a discussion of articular-geometry-inspired coordinate system creation by Kambic et al. [2014]).

To implant spherical markers, expose the bone surface, hand-drill a hole using a drill bit of equivalent diameter to the marker, and insert the marker. Apply a small layer of an adhesive (e.g., cyanoacrylate) over each hole to ensure that all markers stay in place. Conduct practice implantations on additional cadavers (if available) to plan out effective implantation routes and sites before working with study specimens. If bare bone is not visible—for example, in an intact cadaver or reduced preparation with only the integument removed—implant all markers through surgical routes using small incisions and blunt dissection, as if the animal were alive, to minimize damage to soft tissues. Once implantation is complete, any incisions into soft tissues should be sutured to restore the tissues’ original constraints on mobility as much as possible in the absence of healing.

**Fig. 3:**
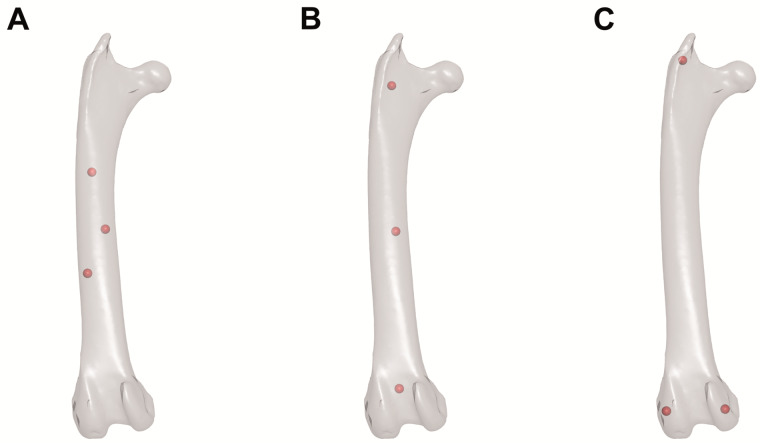
Best practices for XROMM radiopaque marker placement. Hypothetical positions of three radiopaque markers (represented by red spheres) in a CT-derived mesh model of a right avian femur in cranial view. (**A**) Poorly placed markers: too close together. (**B**) Poorly placed markers: too co-linear. (**C**) Well-placed markers: far apart and not co-linear. Note that these practices apply to both *in vivo* and *ex vivo* studies.

Take care to ensure that markers are both (1) implanted as far apart from each other as anatomically possible and (2) not placed co-linearly so that motions in all degrees of freedom can be measured reliably (Brainerd et al. 2010; Fig. 3). If an element is particularly small or slender, fashioning “bead on post” markers by drilling a hole into a spherical marker and inserting a steel post (e.g., cut from an insect pin), and implanting these markers’ posts into the bone, may help to increase inter-marker spacing (Brainerd et al. 2010). In general, using larger radiopaque markers (e.g., 0.8–1.0 mm diameter rather than 0.5 mm) will cause each marker to be represented by more pixels in X-ray videos, improving the performance of the automatic marker detection algorithm used by XMALab (Knörlein et al. 2016). Larger markers are therefore preferable if they will not cause additional soft tissue damage—especially for researchers using X-ray systems with low image resolution. Before beginning data collection, err on the side of caution and collect a reference CT scan of the specimen in case any damage to skeletal elements occurs during manipulation.

## Manipulator attachment

**Fig. 4:**
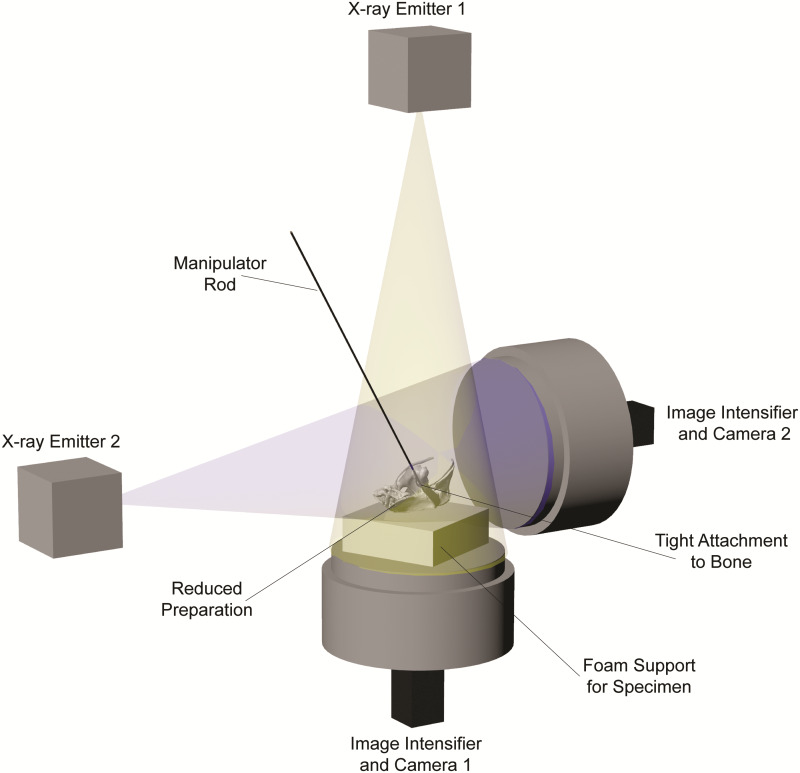
Biplanar X-ray image machine setup for an *ex vivo* joint mobility study using XROMM. A diagrammatic representation of the manipulation of a reduced avian hip joint preparation (see also Fig. 5A). Although this image shows independent X-ray emitters and image intensifiers, the same principles hold for C-arm fluoroscope systems.

After specimens have been marked as fully as possible, they must be manipulated within the volume created by two X-ray image machines to capture biplanar X-ray videos (Fig. 4). The large moment arms created by manipulating a cadaveric joint from a safe distance outside the X-ray volume (i.e., with a long rod) make it easy to accidentally fracture skeletal elements or tear joint soft tissues, especially in small individuals. To prevent this damage, use flexible rather than rigid rods to construct manipulators—it is far easier to replace a broken manipulator than a broken specimen. Wooden, low-diameter (i.e., 3–5 mm) dowel rods sold at craft and hardware stores offer one effective and inexpensive solution. Alternatively, 3D printing or laser-cutting can be used to fashion customized and reproducible manipulators from a variety of pliable materials.

**Fig. 5:**
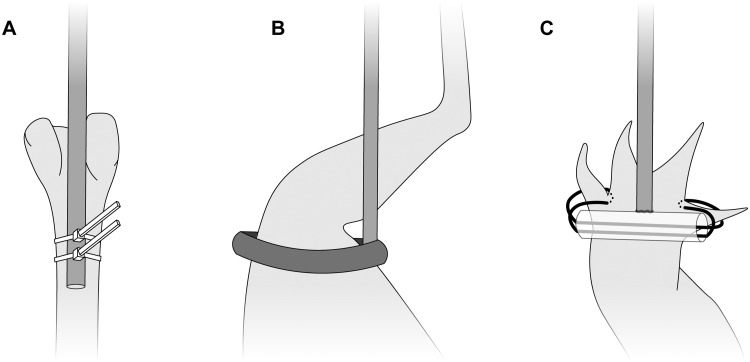
Diagrammatic representation of three potential manipulator attachments. (**A**) Zip ties tightly affix a rod to bare avian femur in a reduced preparation. (**B**) A loose loop of Velcro attached to a rod enables manipulation of an intact avian hindlimb without constricting soft tissues. (**C**) Elastic threaded through the interdigital integument allows minimally invasive attachment of a rod, via a small plastic tube, to an intact crocodylian pes.

Effectively attaching a manipulator rod to the specimen is arguably the most challenging aspect of an *ex vivo* joint mobility study using XROMM. Make sure to use radiolucent attachment materials to avoid complicating radiopaque marker tracking. For studies on reduced preparations with visible regions of bone (e.g., Fig. 4), tightly attach the rod to one of the joint’s mating bones using string or plastic cable ties (Fig. 5A; following Kambic et al. 2017a; Manafzadeh and Padian 2018). Fashioning attachments for intact cadavers or reduced preparations with no visible bone is much more difficult because the attachment should never constrict soft tissues and alter their effects on mobility (*contra*Arnold et al. 2014; Kambic et al. 2017b). For limb joints, consider loosely attaching the rod using elastic and/or slack rings of Velcro (Fig. 5B). Alternatively, for cranial or axial joints, or for attachments to the manus or pes, consider attaching the rod by threading loops of suture or elastic through the integument (only if it is thick enough to resist the forces of manipulation; e.g., tough reptile skin; Manafzadeh et al. under review), taking care not to damage the underlying muscle (Fig. 5C). When manipulating intact multi-joint chains such as limbs, attaching two manipulator rods simultaneously—one more proximally and one more distally—may be necessary to gain sufficient control over the joint(s) of interest.

Ultimately, the process of manipulator attachment is highly organism- and joint-specific and requires both a healthy dose of creativity and a lengthy process of trial and error. Do not underestimate this step of the workflow. Before beginning an *ex vivo* mobility study using XROMM, thoroughly consider whether manipulator rods can be attached to the joint(s) of interest without damaging or constricting the tissue(s) of interest, and in turn, whether the desired ROM study is actually viable. Always build and test manipulators and confirm their effectiveness well before entering the X-ray lab for data collection.

## X-ray data collection

Begin X-ray data collection by taking still images of a standard grid and object of known geometry to allow undistortion of fluoroscopic videos and calibration of the X-ray cameras (see Knörlein et al. 2016). Once a manipulator rod has been attached to the specimen, mount the specimen on foam (or another radiolucent material) to raise it to the center of the X-ray imaging volume, in view of both X-ray cameras. If using a dorsoventral–mediolateral X-ray image machine configuration, foam can be placed directly on top of the dorsoventral image intensifier (Fig. 4). If using an oblique configuration instead, construct a foam-topped support structure of appropriate height using crates, boxes, or other materials, making sure to keep radiopaque elements of the structure outside the X-ray volume. Keep in mind that each element of hardware introduced into the setup may create a new obstruction for effective manipulation of the specimen (as will the X-ray emitters and image intensifiers themselves). After ensuring that the region of interest is in view of both X-ray cameras, loosely affix the specimen to the foam (again to introduce some flexibility into the system and avoid breaking the specimen when applying large forces; see Manipulator attachment section), and firmly affix the foam to the support structure or image intensifier, using loops of tape.

When manipulating the specimen to collect X-ray videos, make sure to move the joint slowly and smoothly enough, and to use a frame rate that is high enough (i.e., 50 fps or higher), to allow XMALab software (Knörlein et al. 2016; see Marker tracking and animation section) to automatically track the 2D positions of the radiopaque markers. It may be helpful to conduct a single test trial and attempt to analyze the resulting videos in XMALab to ensure that the combination of imaging settings and manipulation speed allows effective automatic tracking within the software; making any necessary experimental setup adjustments at this stage can save hundreds to thousands of hours of manual analysis time. Once the effectiveness of the experimental setup has been confirmed, conduct many trials to amass a dense, complete sample of joint poses. At 50 fps, no fewer than 10,000 total video frames, but ideally at least 20,000, should be collected (see a sensitivity analysis of frame count effects on rotational mobility computations in Manafzadeh and Gatesy 2020); a higher frame rate will require proportionally more video frames to be collected and analyzed to achieve the same sampling of full mobility. Throughout this process, reorient the specimen (if possible) and re-position the manipulator attachment several times to ensure that elements of the experimental setup are not inadvertently blocking any joint poses. If a short break is necessary during data collection, tightly seal the specimen in a plastic bag and store it in a refrigerator to prevent further deterioration or desiccation of soft tissues. Make sure to regularly irrigate reduced preparations with Ringer’s solution or physiological saline throughout the study.

Using a pulsed X-ray generation mode (e.g., 2 ms pulse width) will help to limit the amount of radiation produced, and placing lead shields around the X-ray image machines and wearing X-ray safety apparel will help to further limit the amount of radiation that reaches the researcher conducting the manipulation. If they are used, ensure that lead shields are strategically placed so that they do not interfere with the researcher’s ability to manipulate the specimen. Once video data collection is complete, once again take still images of a standard grid and object of known geometry to allow accurate undistortion of fluoroscopic videos and calibration of the X-ray cameras for all trials. This additional set of images is essential if any element of the setup was shifted (intentionally or unintentionally) throughout the day.

Carefully dissect the specimen under a dissecting microscope to assess whether all articular structures have remained fully intact. If any tissues that were meant to remain intact are damaged, then the specimen’s data may or may not be salvageable depending on whether it can be determined when the damage occurred (see Marker tracking and animation section). Fully disarticulate the specimen (if necessary) and obtain a final CT scan. Then, generate polygonal mesh models of radiopaque markers and skeletal elements for use in creating XROMM animations. Finally, store all resulting data and metadata in a repository such as the X-ray Motion Analysis Portal (XMAPortal; xmaportal.org), following the best practices for video data management outlined by Brainerd et al. (2017).

## Marker tracking and animation

The development of XMALab software (Knörlein et al. 2016) has substantially streamlined the process of X-ray video analysis since the *in vivo* marker-based XROMM workflow was originally published by Brainerd et al. (2010). Use XMALab to correct fluoroscope distortion, calibrate the X-ray cameras, track the 2D positions of radiopaque markers in both videos, and calculate RBTs for each skeletal element. Unfortunately, the deep learning tracking package DeepLabCut does not currently perform well in facilitating marker tracking for comprehensive ROM studies (Laurence-Chasen et al. 2020), though future development may allow more successful integration of XMALab and DeepLabCut for cadaveric manipulations. For an overview of and guide to using XMALab, see both the original publication (Knörlein et al. 2016) and the XROMM website (xromm.org).

Because cadaveric manipulations are largely acyclic, do not apply XMALab’s low-pass Butterworth filter to the RBTs (Knoerlein, pers comm). Instead, carefully refine tracking using XMALab’s plots for reprojection error and rigid body error. After refinement, check XMALab’s six-degree-of-freedom RBT plot for any remaining outliers, and then export the fully refined, unfiltered RBTs. As in any XROMM study, the freely available XROMM Tool Shelf (xromm.org) for Autodesk Maya (Autodesk, San Rafael, CA) can be used to animate CT-derived mesh models of the skeletal elements with the exported RBTs. Alternatively, custom Maya Embedded Language or Python scripts can be written to accomplish this task within Maya, or alternatives to Maya altogether, such as the svgViewR R package (Olsen 2018; e.g., Olsen et al. 2019) can be used to generate animations instead. Then, create taxon-specific JCSs (*sensu*Grood and Suntay 1983) following existing XROMM studies or the general principles embodied by Grood and Suntay (1983) and Wu et al. (2002, 2005), and use the Output Relative Motion XROMM Shelf tool or custom scripts to calculate six-degree-of-freedom kinematics for each joint of interest.

**Fig. 6:**
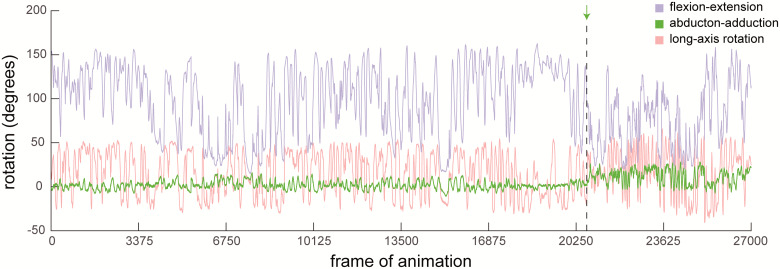
A sudden increase in the magnitude of a degree of freedom’s excursions indicates damage to soft tissues. Rotational kinematics of the knee of an intact Helmeted Guineafowl (*N. meleagris*) cadaver. Dashed line indicates where damage likely occurred, as evidenced by a sudden and substantial increase in excursions in ABAD (green). All data from this trial and beyond should be eliminated before computing mobility.

Using the Maya Graph Editor (or alternatively, Matlab [Mathworks, Natick, MA], R [R Foundation for Statistical Computing, Vienna, Austria], Excel [Microsoft Corporation, Redmond, WA] or another external graphing software), produce kinematics versus time graph and evaluate whether any rotational or translational degree of freedom demonstrates a sudden increase in the magnitude of its excursions (i.e., a suddenly larger range of values; Fig. 6). If so, the joint likely became damaged at that point in time. Therefore, exclude all data (for the whole specimen, even if more than one joint was marked, because the cadaver is no longer intact) from the trial where the increase in magnitude occurs until the end of the study. This exclusion is critical because the joint poses measured during these trials do not reflect the study’s intended ROM. If soft tissue damage was noted during the post-manipulation dissection (see X-ray data collection section), but no clear shift is visible in the kinematics versus time graphs, then it is not possible to determine when the specimen was damaged, and all data from that specimen must, unfortunately, be excluded from analysis.

## Mobility computation

Unfortunately, no metric has yet been created to quantify six-degree-of-freedom (synthesizing translational and rotational data) joint mobility. However, the measurement of joint rotational mobility—incorporating data from all three rotational degrees of freedom—has been refined substantially over the past decade. Whereas traditional ROM studies reported maximum excursions possible in each rotational degree of freedom separately, typically as bar graphs and/or tables, recent studies have found that joints demonstrate substantial interactions among rotational degrees of freedom (e.g., Haering et al. 2014; Kambic et al. 2017b; Manafzadeh and Padian 2018; Manafzadeh et al. under review; see an extended discussion by Haering et al. [2014] and Kambic et al. [2017a]). In other words, joints cannot typically reach maximum excursions in all their rotational degrees of freedom simultaneously. This finding reinforces the value of an XROMM-based approach to studying joint mobility and implies that computations of rotational mobility must be conducted in 3D to be biologically meaningful.

Several more recent studies (e.g., Haering et al. 2014; Kambic et al. 2017a; Manafzadeh and Padian 2018) have thus plotted all measured joint poses as 3D points in an “Euler space” whose three axes are the simultaneous excursions measured in each of a joint’s rotational degrees of freedom, as follows:

$$\begin{array}{c} X_{\text{EULER}}=\alpha \\ Y_{\text{EULER}}=\beta \\ Z_{\text{EULER}}=\gamma \end{array}$$

where α is the angle measured about the first JCS axis in the rotation sequence, β is the angle measured about the second JCS axis in the rotation sequence, and γ is the angle measured about the third JCS axis in the rotation sequence. For most (but not all) comparative XROMM studies to date, these correspond to *Z* (e.g., flexion–extension), *Y* (e.g., abduction–adduction), and *X* (e.g., long-axis rotation) rotations, respectively. Once all joint poses are plotted in this space, the full set of points is then “shrink-wrapped” using a convex hull or other alpha shapes, and the volume of this polygonal envelope is computed as a metric for rotational mobility.

However, Manafzadeh and Gatesy (2020) determined that volumes measured in Euler space are both non-linearly distorted and coordinate-system-dependent. As a result, they created a “cosine-corrected Euler space” to resolve this distortion and allow coordinate-system-independent measurements and comparisons of joint rotational mobility. The equations necessary to plot poses in this space were first proposed by Manafzadeh and Gatesy (2020) and are reproduced here:

$$\begin{array}{c} X_{\text{CC}}=(\alpha -\alpha_{\text{central}})\;\text{cos}(\beta )\\ Y_{\text{CC}}=\beta \\ Z_{\text{CC}}=\gamma \end{array}$$

where α, β and γ are defined as above, and α_central_ is the α value selected to be centered in the space (analogous to selecting the prime meridian to be centered on a 2D map of the world).

**Fig. 7:**
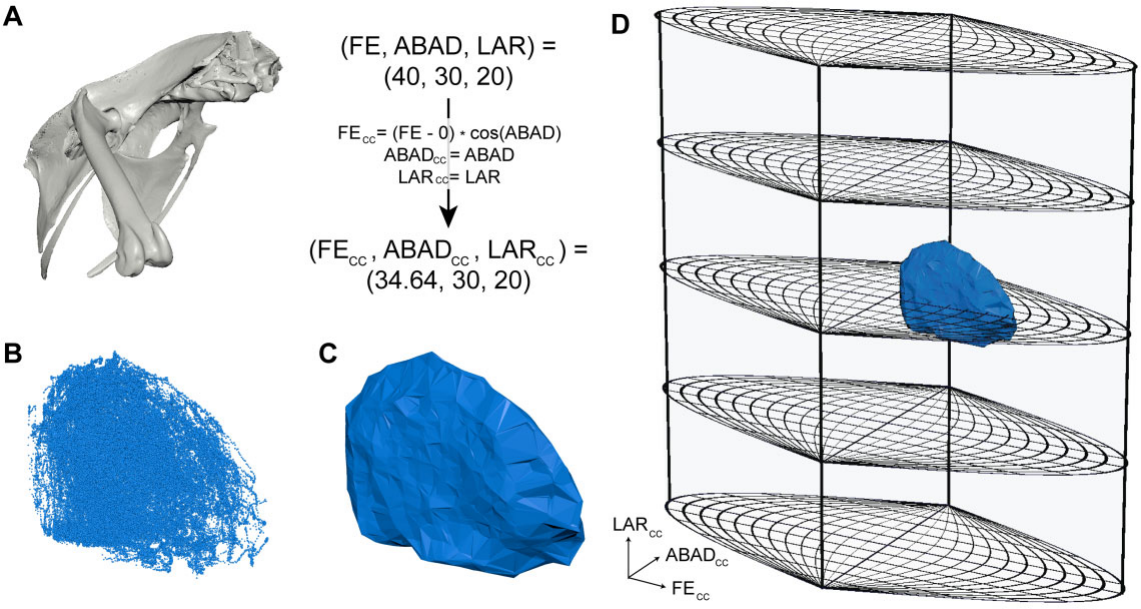
Compute rotational mobility by plotting joint poses in cosine-corrected Euler space. Data are poses measured from a guineafowl hip. (**A**) Every joint pose (here displayed on a right avian hip joint viewed anterolaterally) can be converted from a triple of Euler angles measured using a JCS to a point in cosine-corrected Euler space. FE, flexion–extension; ABAD, abduction–adduction; LAR, long-axis rotation. Here α_central_ is set to 0 as in Manafzadeh and Gatesy 2020. (**B**) Plotting all cosine-corrected joint poses creates a point cloud. (**C**) An alpha shape can then be calculated for the point cloud, yielding a polygonal envelope that represents the joint’s full ROM. (**D**) These ROM envelopes can be viewed in the context of a full cosine-corrected Euler space, enabling ROM mapping comparisons (see Manafzadeh and Padian 2018).

Therefore, to compute joint rotational mobility from XROMM ROM data, begin by plotting all joint poses in cosine-corrected Euler space (If joint poses were measured using a proper Euler rather than Tait-Bryan JCS [e.g., for studies of human shoulders; see Wu et al. 2005], use sine-corrected Euler space instead; see Manafzadeh and Gatesy 2020). If the joint poses are split into two or more clusters due to the aliasing of Euler angles, change the value of *α*_central_ to shift all the poses along the *α* axis and unite the pose clusters as fully as possible. Then, fit an alpha shape (with an alpha radius of 10 or the critical alpha radius, whichever is greater; see Manafzadeh and Gatesy 2020) to the resulting point cloud and measure its volume. This procedure can be done in Matlab (Mathworks, Natick, MA; see alpha shape computation code as part of the Electronic Supplementary Material to Manafzadeh and Padian 2018) but can also be done using any programming language that allows the computation of alpha shapes (see Fig. 7).

For studies aiming to compare the mobility of a joint with different degrees of soft tissue intact, the same specimen should be used for all manipulations. In the same vein, when aiming to compare true mobility to the poses used in life, it is best to manipulate the intact cadaver of a specimen previously used in an *in vivo* XROMM study. Of course, this consistency is not always practical, but whenever possible conducting subject-specific comparisons circumvents slight discrepancies in poses measured from different joints, which will exist—even when coordinate systems are created in a standardized and reproducible way—due to anatomical variation (see Kambic et al. 2017a; Manafzadeh and Padian 2018 for examples of intraspecific, inter-individual comparisons). If, for whatever reason, it is not possible to re-use the same individual, increase the specimen sample size to account for inter-individual variation. Each joint included adds tens thousands of additional frames that must be analyzed, which can quickly limit the feasibility of a study. However, including at least three total joints from at least two individuals will enable basic analyses of intraspecimen and interspecimen variation, and can help to reveal if any of the joints (or individuals) studied is significantly different from the others. If this appears to be the case, the sample size must be further increased to increase statistical power and allow quantitative tests of whether one joint or one individual is truly an outlier.

## Concluding remarks

Using XROMM to conduct an *ex vivo* study of joint mobility may, at first, seem fundamentally easier than conducting an *in vivo* study. After all, there are no survival surgeries to conduct and no live animals to train. However, these studies present their own set of challenges—ones that require the same levels of creativity and patience to overcome as those of *in vivo* experiments. When executed well, XROMM studies of joint mobility have the potential to substantially advance our knowledge of articular function. They facilitate an unprecedented look inside joints in all their possible poses, producing data that are relevant to the work of both neontologists and paleontologists alike. Although future improvements to these existing methods are both likely and extremely welcome, the framework provided by this article will continue to serve as a general guide to conducting ROM studies with XROMM.
